# A transgenic bacterial artificial chromosome approach to identify regulatory regions that direct *Amhr2* and *Osterix* expression in Müllerian duct mesenchyme

**DOI:** 10.3389/fcell.2022.1006087

**Published:** 2022-10-12

**Authors:** Malcolm M. Moses, Rachel D. Mullen, Daniel I. Idowu, Peter Maye, Soazik P. Jamin, Richard R. Behringer

**Affiliations:** ^1^ Department of Genetics, University of Texas MD Anderson Cancer Center, Houston, TX, United States; ^2^ Graduate Program in Genetics and Epigenetics, MD Anderson Cancer Center UTHealth Graduate School of Biomedical Sciences, Houston, TX, United States; ^3^ Department of Reconstructive Sciences, School of Dental Medicine, University of Connecticut Health Center, Farmington, CT, United States; ^4^ Université de Rennes, Inserm, EHESP, Irset (Institut de Recherche en Santé, Environnement et Travail), Rennes, France

**Keywords:** osterix, osx-cre, Sp7/osterix, anti-müllerian hormone receptor 2, transgenic mice, sex diffentiation, bacterial artificial chromosome (BAC)

## Abstract

A transgenic mouse approach using bacterial artificial chromosomes (BAC) was used to identify regulatory regions that direct Müllerian duct expression for *Amhr2* and *Osterix* (*Osx*, also known as *Sp7*). *Amhr2* encodes the receptor that mediates anti-Müllerian hormone (AMH) signaling for Müllerian duct regression in male embryos. *Amhr2* is expressed in the Müllerian duct mesenchyme of both male and female embryos. A ∼147-kb BAC clone containing the *Amhr2* locus was used to generate transgenic mice. The transgene was able to rescue the block in Müllerian duct regression of *Amhr2*-null males, suggesting that the BAC clone contains regulatory sequences active in male embryos. *Osx* is expressed in the developing skeleton of male and female embryos but is also an AMH-induced gene that is expressed in the Müllerian duct mesenchyme exclusively in male embryos. *Osx-Cre* transgenic mice were previously generated using a ∼204-kb BAC clone. Crosses of *Osx-Cre* mice to Cre-dependent *lacZ* reporter mice resulted in reporter expression in the developing skeleton and in the Müllerian duct mesenchyme of male but not female embryos. *Osx-Cherry* transgenic mice were previously generated using a 39-kb genomic region surrounding the *Osx* locus. *Osx-Cherry* embryos expressed red fluorescence in the developing skeleton and Müllerian duct mesenchyme of male but not female embryos. In addition, female *Osx-Cherry* embryos ectopically expressing human AMH from an *Mt1-AMH* transgene activated red fluorescence in the Müllerian duct mesenchyme. These results suggest that the 39-kb region used to generate *Osx-Cherry* contains male-specific Müllerian duct mesenchyme regulatory sequences that are responsive to AMH signaling. These BAC transgenic mouse approaches identify two distinct regions that direct Müllerian duct mesenchyme expression and contribute fundamental knowledge to define a gene regulatory network for sex differentiation.

## Introduction

Mammalian reproductive tract organ development begins with the formation of two genital ducts within the paired mesonephroi (Mullen and Behringer, 2014). The embryo, regardless of its sex genotype, first forms the Wolffian or mesonephric ducts, which have the potential to develop into male reproductive tract organs, including the epididymides, vasa deferentia, and seminal vesicles. Subsequently, the Müllerian or paramesonephric ducts form along the Wolffian ducts (Orvis and Behringer, 2007). The Müllerian ducts have the potential to develop into female reproductive tract organs, including the oviducts, uterus, cervix, and upper vagina. The elongation of the Müllerian ducts to the urogenital sinus in male and female mouse embryos is complete by E13.5 (Orvis and Behringer, 2007). During sex development, one pair of the genital ducts (Wolffian or Müllerian) will differentiate and the other will be eliminated. This is regulated by hormones and growth factors produced from the fetal testes or not produced by the fetal ovaries.

In male embryos, the testes send a signal to the Müllerian ducts, which triggers Müllerian duct regression, causing gaps in the Müllerian duct epithelium that lead to its elimination. This signal comes in the form of anti-Müllerian hormone (AMH) (Cate et al., 1986; Picard et al., 1986; Josso et al., 1993; Behringer, Finegold and Cate, 1994). The primary receptor that receives AMH in the Müllerian duct, anti-Müllerian hormone receptor 2 (AMHR2), forms a receptor complex with Type I receptors to trigger regression (Mishina et al., 1996, 1999; Gouedard et al., 2000; Clarke et al., 2001; Visser et al., 2001; Belville et al., 2005; Zhan et al., 2006; Orvis et al., 2008). *Amhr2* is expressed in the Müllerian duct mesenchyme of both male and female embryos, indicating that the mesenchyme is the target tissue of AMH and that Müllerian duct regression results from mesenchyme-epithelial interactions (Baarends et al., 1994). Indeed, ectopic expression of AMH from an *Mt1-AMH* transgene induces Müllerian duct regression in females, resulting in the absence of oviducts and uterus (Behringer et al., 1990). The AMH receptor complex signals through redundant *Smad* genes (Orvis et al., 2008). Following this initial signal is a cascade of genetically regulated activity for Müllerian duct regression that has yet to be fully elucidated. However, a gene regulatory network has been described primarily based on genetic loss-of-function studies of Müllerian duct regression (Moses and Behringer, 2019).

A recent addition to the gene regulatory network for Müllerian duct regression is *Osterix* (*Osx*), formally known as *Sp7* (Mullen et al., 2018). *Osx* encodes a zinc-finger transcription factor that is essential for osteoblast and odontoblast differentiation (Nakashima et al., 2002; Bae et al., 2018). Previously, we showed that *Osx* is an AMH-induced gene that is expressed in the Müllerian duct mesenchyme (Mullen et al., 2018). This places *Osx* downstream of *Amhr2* in the gene regulatory network for Müllerian duct regression (Moses and Behringer, 2019). In contrast to *Amhr2* which is expressed in the Müllerian duct mesenchyme of both male and female embryos, *Osx* is expressed in the Müllerian duct mesenchyme only in male embryos. Intriguingly, *Amhr2* and *Osx* are located within 100 kb of each other.

Enhancers are DNA sequences that are bound by proteins to form complexes at basal promoters to direct cell type and tissue-specific transcription (Field and Adelman, 2020). These DNA sequences can reside close to a locus (within 1 kb) or can be located at relatively large distances (∼1 Mb) (Lettice et al., 2003). Enhancers have been identified 5′ to a locus, within introns, or 3’ to a locus (Ornitz et al., 1985; Behringer et al., 1987; Mukhopadhyay et al., 1995). Classically, enhancers have been identified using *in vitro* assays or *in vivo* using transgenic animals. More recently, analysis of chromatin modifications and open chromatin domains have identified candidate transcriptional enhancers (Vu and Ernst, 2022). Ultimately, enhancers that can direct tissue-specific transcription must be tested for activity *in vivo*. Although cis-regulatory elements in the gene regulatory network for Müllerian duct regression have been identified for the *Amh* gene expressed in the Sertoli cells of the testes, no cis elements have been identified for Müllerian duct epithelium or mesenchyme transcription. Previously, multiple lines of *Amhr2-EGFP* transgenic mice were generated using a 500-bp promoter region but reporter expression was not detected (Kimura et al., 2017). Thus, the immediate upstream region of *Amhr2* is not sufficient for cell type-specific transcription.

In this study, we sought to identify regulatory regions associated with *Amhr2* and *Osx* that direct Müllerian duct mesenchyme transcription, using a bacterial artificial chromosome (BAC) transgenic mouse approach. We screened BAC clones containing *Amhr2* or *Osx* for Müllerian duct mesenchyme-specific transcriptional activity in transgenic mice. Using this assay, we have identified two genomic regions that direct Müllerian duct mesenchyme-specific activity. The subsequent identification of Müllerian duct-specific regulatory regions will further define the gene regulatory network for Müllerian duct regression during male differentiation.

## Materials and methods

### Mice

Swiss outbred mice were purchased from Taconic Biosciences. B6SJLF1/J and *Tg(Sp7-tTA,tetO-EGFP/cre)1Amc (Osx-Cre)* transgenic mice were obtained from the Jackson Laboratory. *Tg(Sp7/mCherry)2Pmay/J (Osx-Cherry)* transgenic mice were produced using a CD1 outbred stock (Strecker et al., 2013). *Osx-Cre* and *Osx-Cherry* mice were subsequently outcrossed to Swiss Webster mice (Taconic Biosciences). *Amhr2* ^*ΔE1-6*^ (*Amhr2-ΔE1-6*; Mishina et al., 1996), *Amhr2* ^*tm2Bhr*^ (*Amhr2-lacZ*; Arango et al., 2008), *Gt(ROSA)26Sor* ^*tm1(lacZ)Sor*^ (*R26R-lacZ*; Soriano 1999), and *Mt1-AMH* (Behringer et al., 1990) mice were maintained on a predominantly C57BL/6J genetic background. All animal procedures were approved by the Institutional Animal Care and Use Committee of the University of Texas MD Anderson Cancer Center. Studies were performed consistent with the National Institutes of Health Guide for the Care and Use of Laboratory Animals.

### Generation of *Amhr2* BAC transgenic mice

High density BAC clone arrays on filters from the RPCI-22 129/SvEv female mouse genomic library (BACPAC Genomics, Emeryville, CA) were screened with a^32^P-labelled probe located ∼7 kb 5′ of *Amhr2* exon 1, using a Megaprime DNA Labelling System kit (Amersham) (Mishina et al., 1996). Six positive clones were identified using the manufacturer’s grid key. Purified BAC clone DNA was digested with NotI, subjected to pulse-field agarose gel electrophoresis, and analyzed by Southern blot, using the same probe used in the initial screen. An additional probe within intron 6 of the *Amhr2* locus was used (Mishina et al., 1996). This analysis suggested that all six BAC clones contained the *Amhr2* locus. Sequencing of the ends of BAC clone RPCI-22 315D12 delineated the genomic region contained in the clone (NCBI37/mm9: Chr 15, 102,401,136–102,548,427). RPCI-22 315D12 was linearized with I-SceI. The linearized DNA (0.2 ng/ul) was injected into the pronuclei of B6SJLF2/J hybrid zygotes and then transferred into the oviducts of pseudopregnant Swiss surrogate mothers.

### Genotyping

*Osx-Cre, Osx-Cherry*, *Amhr2* ^*ΔE1-6*^, *Amhr2-lacZ*, *R26R-lacZ* were genotyped as previously described (Mishina et al., 1996; Soriano 1999; Rodda and Mcmahon, 2006; Arango et al., 2008; Strecker et al., 2013). *Mt1-AMH* transgenic mice were genotyped by PCR using the following primers: hMIS-Fw1: 5′ CCC TAG TGC TGT CTG CCC T 3′ and hMIS-Rv2: 5′ GGA GCT GCT GCC ATT GCT G, resulting in a 176 bp amplified DNA fragment. PCR genotyping for the *Amhr2* BAC transgene was performed using the following pBACe3.6 vector primers: BAC-F: 5′ GTG ATA TCG CGG AAG GAA AA 3′ and BAC-R: 5′ AGG ATA TAC GGC AGG CAT TG 3’, resulting in a 499 bp amplified DNA fragment.

### X-gal staining

Embryos were stained for *lacZ* expression as described (Arango et al., 2008).

### Histology

Ten µm sections were cut from paraffin embedded *lacZ*-stained tissues and counterstained with 0.1% Nuclear Fast Red. 10 µm frozen sections were cut from OCT embedded *Osx-Cherry* embryos and stained with DAPI (4′,6-diamidino-2-phenylindole).

### Fluorescent microscopy

Embryos were dissected in phosphate-buffered saline and visualized using a Leica MZ10F fluorescent dissecting microscope for Cherry fluorescence. Images were captured using a JENOPTIK GRYPHAX camera. Frozen sections of *Osx-Cherry* embryos were visualized for Cherry and DAPI fluorescence using an A1 Nikon confocal microscope.

## Results

### Rescue of Müllerian duct regression in *Amhr2*-null males by an *Amhr2* BAC transgene

**FIGURE 1:**
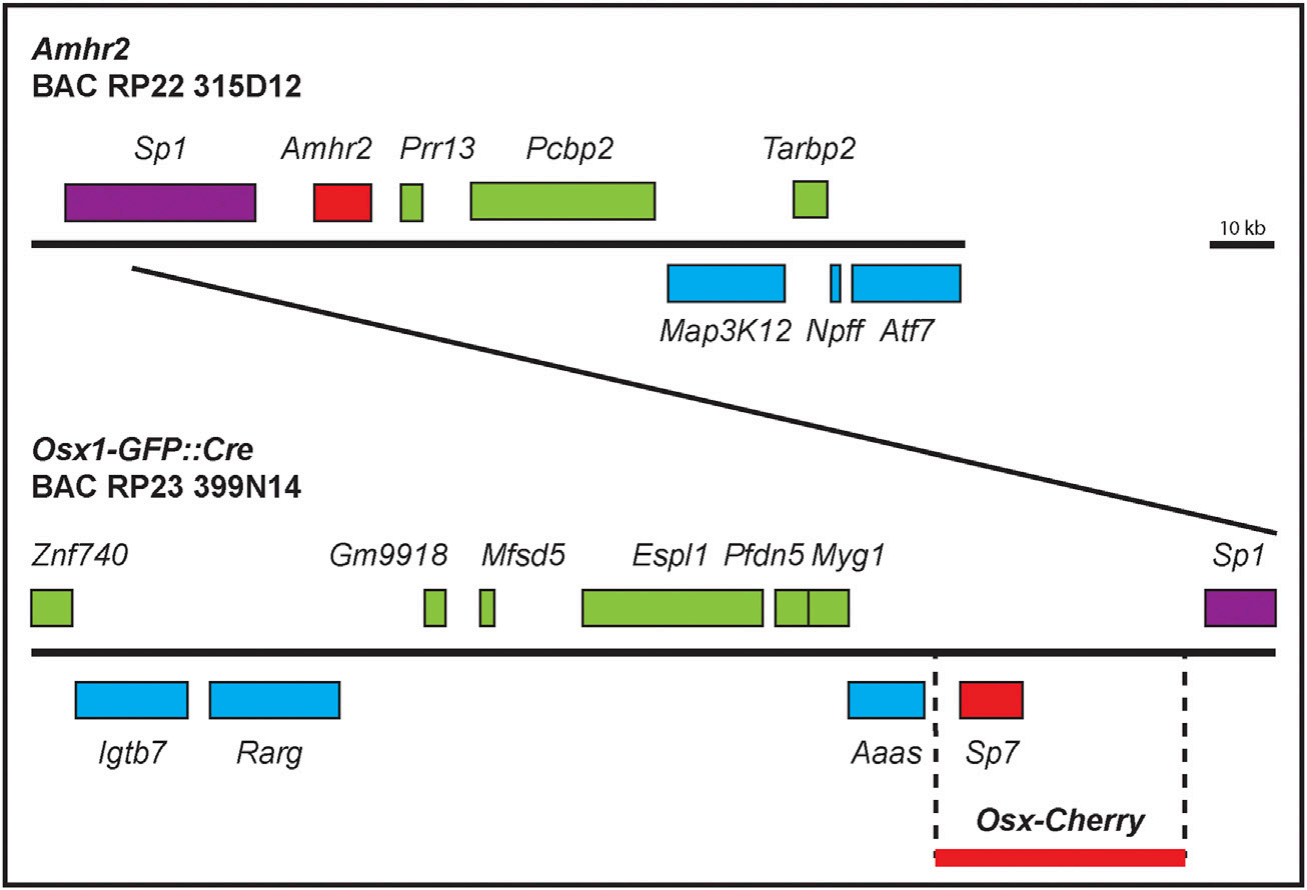
*Amhr2*-and *Osx*-containing BAC clone maps. Diagram of the genomic regions contained within BAC clones RPCI-22 315D12 and RP23-399N14. Genes (introns and exons) are represented by boxes above or below the line, representing the location of the genes on the two strands of DNA. The ∼39 kb region used to create *Osx-Cherry* mice is also shown (thick red line) that lacks coding sequences from neighboring genes.

The lack of cell type-specific regulatory sequences in the 500-bp 5′ region of *Amhr2* (Kimura et al., 2017) motivated us to screen a larger region surrounding the locus. BAC RPCI-22 315D12 has a ∼147 kb (147,291 bp) region of mouse chromosome 15 from a 129S6/SvEvTac inbred mouse, containing the *Amhr2* locus (Figure 1). There is ∼35 kb of sequence 5′ and ∼94 kb 3′ of the *Amhr2* locus. The BAC clone contains 7 other genes (Figure 1). We hypothesized that *Amhr2* Müllerian duct mesenchyme-specific regulatory sequences were located within this BAC clone. To test this idea, we generated transgenic mice with this unmanipulated *Amhr2*-containing BAC clone. Two independent transgenic mouse lines (*Tg* ^*BAC-Amhr2*^) were established.

**FIGURE 2:**
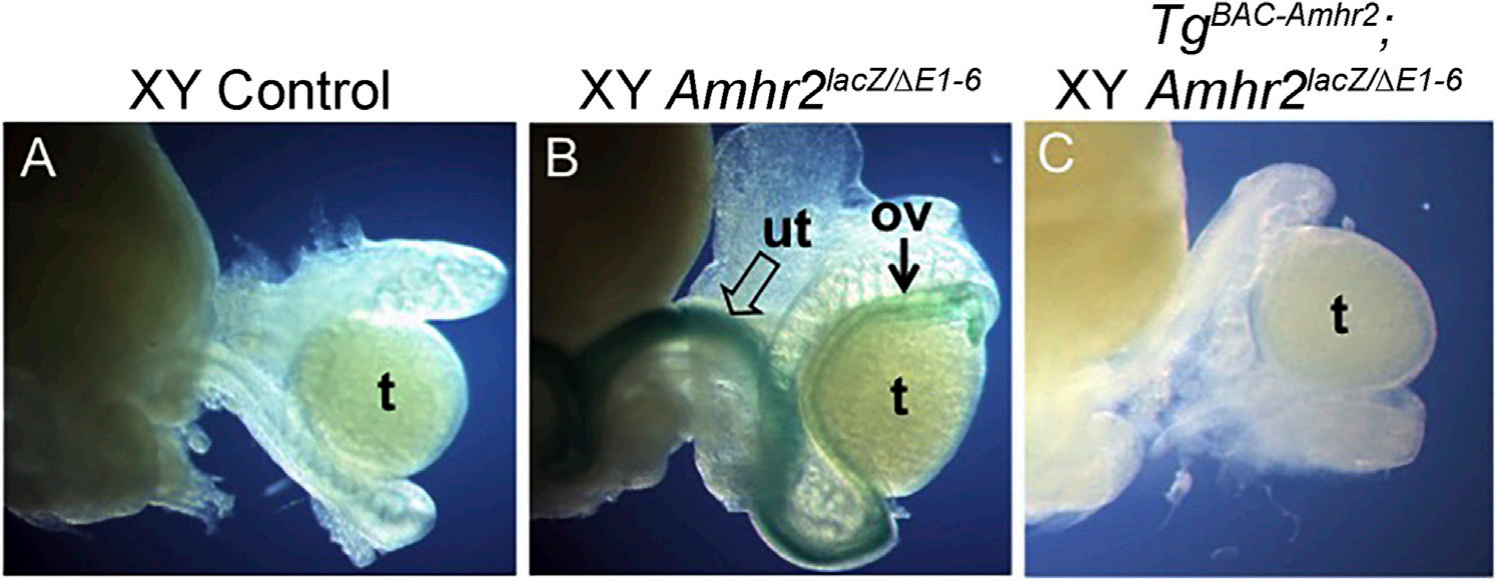
An *Amhr2*-containing BAC transgene rescues the block in Müllerian duct regression of *Amhr2*-null male mice. **(A)** Control male mouse possesses only male reproductive tract organs (epididymis, vas deferens) seen adjacent to the testis. **(B)** *Amhr2* ^*ΔE1-6/lacZ*^ (null) male mouse carry one deletion allele and one *lacZ* knock-in/knockout allele. *lacZ* expression marks the uterus (open arrow) and oviduct (arrow) that forms in these mutants. **(C)** Male mouse with the *Amhr2 BAC* transgene (*Tg* ^*BAC-Amhr2*^) on an *Amhr2* ^*ΔE1-6/lacZ*^ background lacks *lacZ* expression because the block in Müllerian duct regression has been rescued preventing uterus/oviduct differentiation (Line 1, n = 2; Line 2, n = 2). Ov, oviduct; t, testis; ut, uterus.

We next bred male mice from the two *Amhr2* BAC transgenic lines to female mice heterozygous for an *Amhr2-lacZ* allele that is also a null allele (Arango et al., 2008). The *Amhr2-lacZ* allele encodes a convenient visual marker of the uterus and oviduct because *lacZ* is expressed in the myometrium and myosalpinx, respectively (Arango et al., 2008). From the progeny of that cross, *Tg* ^*BAC-Amhr2*^; *Amhr2* ^*lacZ/*+^ male mice were identified and then bred to female mice homozygous for an *Amhr2* deletion allele that is also a null allele (*ΔE1-6*; Mishina et al., 1996) to generate *Tg* ^*BAC-Amhr2*^ transgenic males on an *Amhr2* compound heterozygous null (*ΔE1-6/lacZ*) genetic background. *Amhr2* ^*ΔE1-6/lacZ*^ males served as controls for persistent Müllerian duct derivatives, exploiting the *lacZ* allele to label Müllerian duct derivatives, i. e. uterus and oviducts (Mishina et al., 1996; Arango et al., 2008). At postnatal day 0 (P0) there was complete regression of the Müllerian system in control males (Figure 2A). Positive *lacZ* staining was detected in the persistent uterus/oviduct of an *Amhr2* ^*lacZ/ΔE1-6*^ male (Figure 2B). We found that *Tg* ^*BAC-Amhr2*^; *Amhr2* ^*ΔE1-6/lacZ*^ males were negative for *lacZ* staining because they lacked a uterus and oviduct (Figure 2C). The complete rescue of the *Amhr2* mutant phenotype was observed for both *Amhr2* BAC transgenic lines (Line 1, n = 2; Line 2, n = 2). This suggests that the ∼147 kb *Amhr2*-containing BAC clone contains all of the required regulatory regions for Müllerian duct mesenchyme expression to mediate AMH signaling for regression.

### An *Osx-Cre* BAC transgene activates Cre reporter expression in the Müllerian duct mesenchyme of male embryos

We next used a different BAC transgenic mouse approach to screen for Müllerian duct mesenchyme-specific regulatory regions for *Osx*. BAC RP23-399N14 has a ∼204 kb (204,096 bp) portion of mouse chromosome 15 from a female C57BL/6J mouse containing the *Osx* locus (Figure 1). There is ∼145 kb of sequence 5′ and ∼50 kb 3′ of the *Osx* locus. The BAC clone contains 10 other genes (Figure 1). Like our strategy to identify a Müllerian duct mesenchyme-specific regulatory regions for *Amhr2*, we tested the idea that an *Osx* Müllerian duct mesenchyme-specific regulatory sequences were located within BAC clone RP23-399N14. Transgenic mice carrying this *Osx*-containing BAC have been previously generated (Rodda and Mcmahon, 2006). The *Osx*-containing BAC was previously modified by recombineering to insert a Tet-off regulatable GFP:Cre fusion protein under the control of the *Osx* promoter (*Osx-Cre*). In the absence of doxycycline, Cre expressed from the *Osx* locus mediates recombination of floxed alleles in mice. The *Osx-Cre* BAC transgene was found to be active in the osteoblast lineage throughout embryonic and early postnatal development (Rodda and Mcmahon, 2006).

**FIGURE 3:**
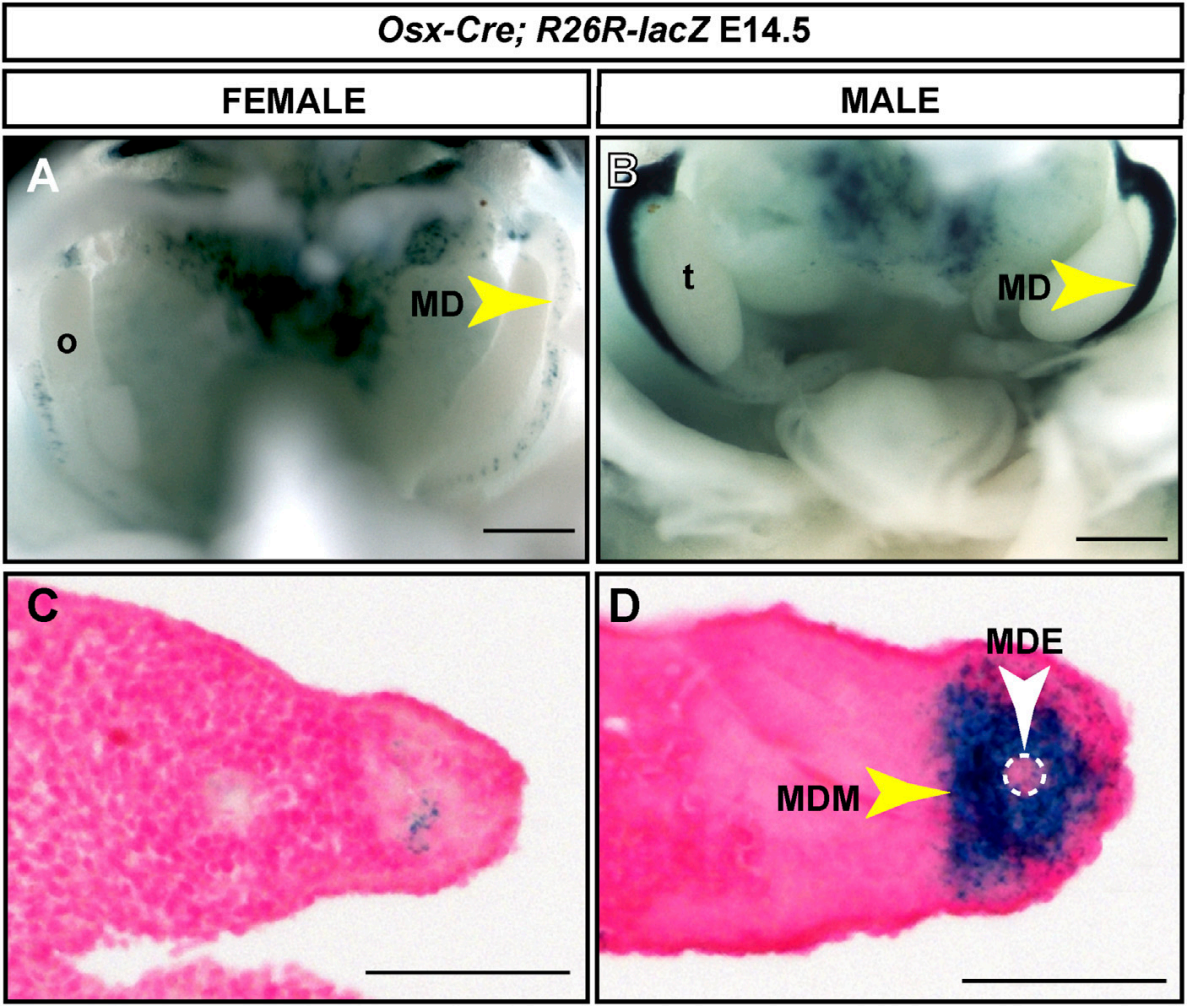
Male-specific Müllerian duct mesenchyme expression induced by an *Osx-Cre* transgene. E14.5 reproductive tract organs from *Osx-Cre tg/0*; *R26R-lacZ*/+ mouse embryos stained for *lacZ* expression. **(A and B)** Whole mount images for female **(A)** and male **(B)** embryos. Scale bar = 500um. **(C and D)** Cross sections through Müllerian ducts counterstained with Nuclear Fast Red. Scale bar = 50 um. t, testis; o, ovary; MD, Müllerian duct; MDE, Müllerian duct epithelium; MDM, Müllerian duct mesenchyme. *Osx-Cre tg/0*; *R26R-lacZ*/+ male embryos, n = 6.

We used the *R26R-lacZ* Cre reporter mouse (Soriano 1999) to screen for *Osx-Cre* expression in the developing Müllerian ducts. Timed matings were established and E13.5 to 16.5 embryos were dissected and stained with X-gal. X-gal staining was detected initially at E14.5 in the developing Müllerian ducts of male embryos (n = 6) (Figure 3). Although *Osx* is expressed throughout the male Müllerian duct mesenchyme (Mullen et al., 2018), we observed strong *lacZ* expression in the anterior Müllerian duct but reduced *lacZ* expression in the posterior Müllerian duct (Figure 3B). X-gal staining was also observed at E15.5 in the male Müllerian duct, in the region adjacent to the testes (n = 4) (data not shown). A very small population of *lacZ*-expressing cells was observed in the Müllerian ducts of female embryos (Figures 3A,C). Histological sections demonstrated that X-gal staining in male embryos was restricted to the Müllerian duct mesenchyme (Figure 3D). This suggests that the ∼204 kb *Osx*-containing BAC contains regulatory information for male-specific Müllerian duct mesenchyme expression.

### A 39 kb region surrounding *Osx* directs male-specific Müllerian duct mesenchyme cherry fluorescent reporter expression in transgenic mice

**FIGURE 4:**
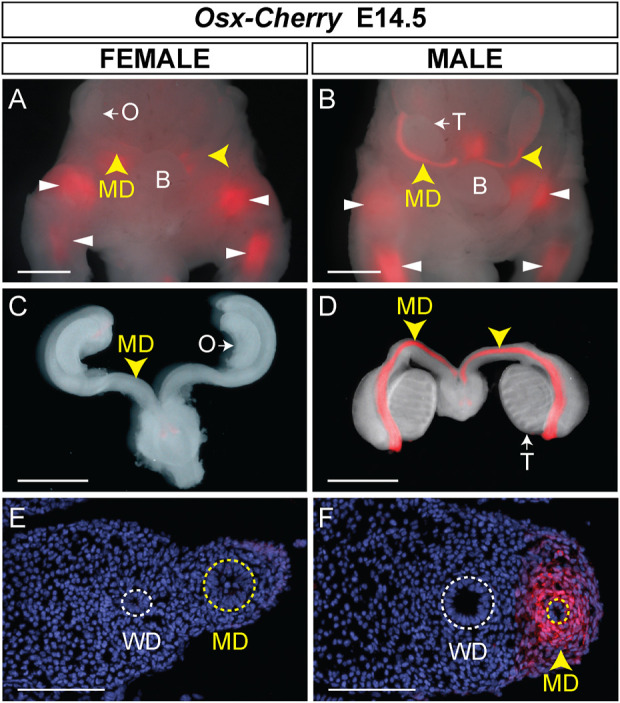
Male-specific Müllerian duct mesenchyme expression in *Osx-Cherry* transgenic mice. **(A–D)** E14.5 reproductive tract organs from *Osx-Cherry tg/0* mouse embryos visualized for Cherry fluorescence. **(A and B)** Ventral views of whole mount brightfield and fluorescent images merged for female **(A)** and male **(B)** embryos. Yellow arrowheads, Müllerian ducts (MD); white arrowheads, skeletal tissues. O, ovary; T, testis. **(C and D)** Brightfield and fluorescent images merged of isolated reproductive tracts at E14.5. **(C)** Female, **(D)** male. **(E and F)** Cross sections through Müllerian ducts, visualized for Cherry fluorescence and counterstained with DAPI. **(E)** Female, **(F)** male. WD, Wolffian ducts. Scale bars **(A–D)** = 1000 um; **(E and F)** = 100 um. *Osx-Cherry tg/0* female embryos, n = 4; male embryos, n = 6.

Transgenic mice have previously been generated using ∼39 kb of genomic sequence containing the *Osx* gene derived from BAC clone RP24-362M3 (C57BL/6J) (Strecker et al., 2013). This ∼39 kb region excluded other gene coding sequences and encompassed ∼20% of the BAC cloned used to generate *Osx-Cre* transgenic mice. A Cherry Fluorescent Protein reporter was inserted just upstream of the second translational start site of *Osx*. Cherry fluorescence was detected in developing and adult skeletal tissues (Strecker et al., 2013). To determine if the ∼39 kb region also contained Müllerian duct mesenchyme regulatory sequences, we examined E14.5 male and female embryos for Cherry fluorescence in the Müllerian ducts (Figure 4). At E14.5, we initially identified *Osx-Cherry* transgenic embryos because they express Cherry fluorescence in the developing skeleton. Male and female *Osx-Cherry* transgenic embryos were identified by brightfield assessment of the gonads. Although Cherry fluorescence was observed in the developing skeleton tissues of female *Osx-Cherry* transgenic embryos, no Cherry fluorescence was detected in the Müllerian ducts (n = 4) (Figures 4A,C). Likewise, Cherry fluorescence was detected in the developing skeleton tissues of male *Osx-Cherry* transgenic embryos (Figure 4B). In addition, Cherry fluorescence was detected in the Müllerian ducts (n = 6) (Figures 4B,D). Histological sections demonstrated that Cherry fluorescence was present in the male but not female Müllerian duct mesenchyme (Figures 4E,F). We also examined AMH-responsive organs, including testes, ovaries and uteri of adult *Osx-Cherry* transgenic and control mice but no Cherry fluorescence was detected compared to controls (Supplemental Figure S1). These results suggest that the ∼39 kb genomic region included in the *Osx-Cherry* transgene contains a male-specific, Müllerian duct mesenchyme regulatory sequences that direct *Osx* transcription.

**FIGURE 5:**
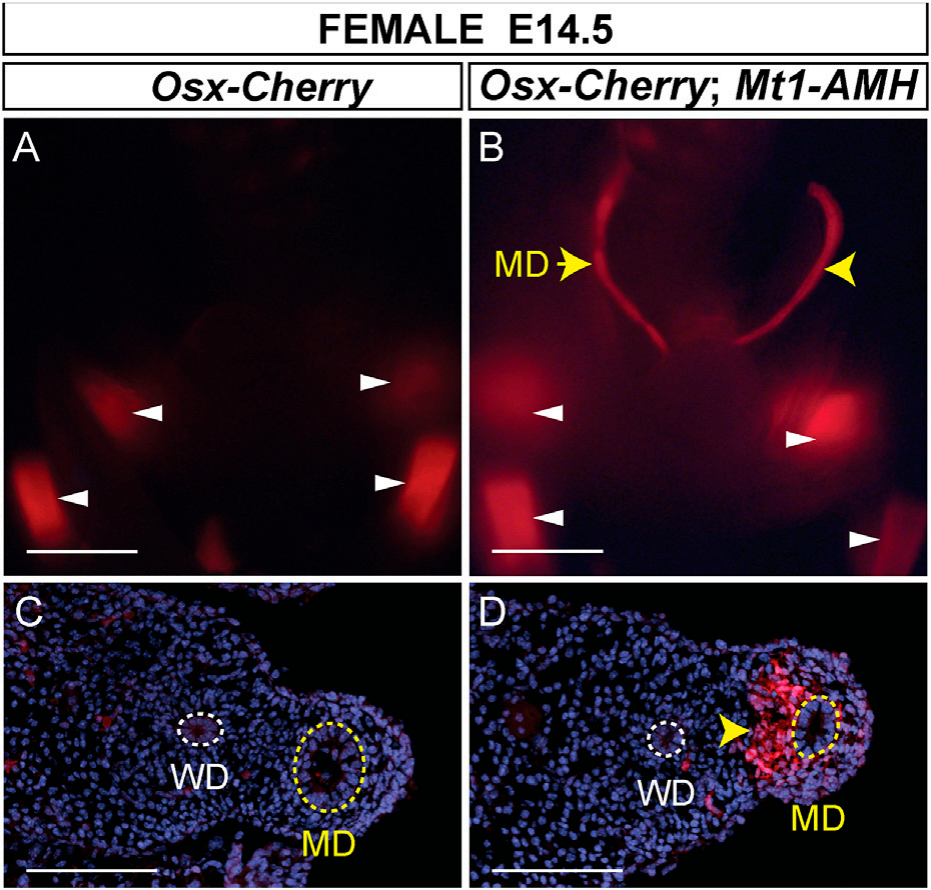
*Mt1-AMH* induces *Osx-Cherry* in the Müllerian ducts of female embryos. **(A and B)** Whole mount images of E14.5 reproductive tract organs from female mouse embryos visualized for Cherry fluorescence. **(A)** *Osx-Cherry tg/0* **(B)** *Osx-Cherry tg/0*; *Mt1-AMH tg/0* female embryos. White arrowheads point to Cherry-expressing bone-forming tissues. Yellow arrowheads point to Cherry expression in the Müllerian ducts. **(C and D)** Cross sections through Müllerian ducts, visualized for Cherry fluorescence and counterstained with DAPI. **(C)** *Osx-Cherry tg/0* **(D)** *Osx-Cherry tg/0*; *Mt1-AMH tg/0* female embryos. Arrowhead in D points to Cherry-expressing Müllerian duct mesenchyme. MD, Müllerian duct (yellow dotted line); WD, Wolffian duct (white dotted line). Scale bars **(A and B)** = 1000 um; **(C and D)** = 100 um. *Cherry tg/0*; *Mt1-AMH tg/0* female embryos, n = 5.

Previously, we showed that an *Osx-lacZ* knock-in allele was expressed in male but not female Müllerian duct mesenchyme, reflecting the sex-specific pattern of *Osx* expression in the Müllerian ducts (Mullen et al., 2018). We also showed that ectopic expression of human AMH from an *Mt1-AMH* transgene could activate the *Osx-lacZ* knock-in allele in female embryos, indicating that *Osx* is an AMH-induced gene (Mullen et al., 2018). To determine if the *Osx-Cherry* transgene could also respond to ectopic human AMH in female embryos, we crossed *Osx-Cherry* females to *Mt1-AMH* males to generate E14.5 *Mt1-AMH*; *Osx-Cherry* double transgenic female embryos (Figure 5). We observed Cherry fluorescence in the Müllerian ducts of the *Mt1-AMH*; *Osx-Cherry* double transgenic female embryos but not in *Osx-Cherry* transgenic female embryos (n = 5) (Figures 5A,B). The Cherry fluorescence in the Müllerian ducts of the *Mt1-AMH*; *Osx-Cherry* double transgenic female embryos was localized to the mesenchyme (Figure 5D). These results demonstrate that the 39 kb *Osx* region contains a Müllerian duct mesenchyme regulatory sequences that are responsive to AMH signaling.

## Discussion

AMH is secreted by Sertoli cells of the fetal testes and subsequently interacts with AMHR2 expressed by the Müllerian duct mesenchyme to induce the elimination of the ductal epithelium, blocking the formation of female reproductive organs in males (Mullen and Behringer, 2014). Previously, we described a gene regulatory network (GRN) for AMH-induced Müllerian duct regression primarily based on *in vivo* genetic data (Moses and Behringer, 2019). Multiple cis regulatory elements have been identified in the 5′ region of the *Amh* gene with binding sites for SOX9, NR5A1, and GATA4 that are required for testis-specific transcription (Shen et al., 1994; De Santa Barbara et al., 1998; Arango et al., 1999; Lasala et al., 2004; Bouchard, et al., 2019). *Amhr2* and *Osx* are expressed in the Müllerian duct mesenchyme for Müllerian duct regression during male differentiation (Baarends et al., 1994; Mishina et al., 1996; Mullen et al., 2018). Although cis regulatory elements have been identified for fetal Sertoli cell-specific expression of *Amh*, no transcriptional enhancers have been identified in the rest of this GRN, notably for genes expressed in the Müllerian duct epithelium or mesenchyme. In this study, we used BAC transgenic mouse approaches to identify genomic regions that contain *Amhr2* and *Osx* sequences that direct Müllerian duct mesenchyme expression.

### Localization of a Müllerian duct mesenchyme regulatory regions for *Amhr2*

We found that transgenic mice generated with a mouse BAC clone containing the entire *Amhr2* locus rescued the persistent Müllerian duct-derived tissues found in *Amhr2*-null males (Mishina et al., 1996; Arango et al., 2008). This suggests that the BAC clone contains regulatory sequences sufficient for Müllerian duct mesenchyme expression. It is also possible that *Amhr2* expression from the BAC clone was expressed ubiquitously. However, this could lead to engagement of Type I receptors for AMH signaling in ectopic tissues that might lead to mutant phenotypes. The *Amhr2* BAC transgenic mice in both lines were normal and fertile.

We also tested the 6.2 kb region 5′ of *Amhr2* including the endogenous promoter for tissue-specific activity using a *lacZ* reporter. However, in 9 independent lines of transgenic mice there was no *lacZ* expression in E14.5 Müllerian ducts of both sexes, adult testes, ovary and uterus (unpublished observations). Thus, the immediate upstream region of *Amhr2* is not sufficient for cell type-specific transcription (Kimura et al., 2017). While our current results are a step forwards, the BAC clone is ∼147 kb in length, creating a challenge to localize the Müllerian duct mesenchyme regulatory sequence to a discreet location.

*Amhr2* transcription in the Müllerian duct mesenchyme is induced by WNT7a secreted by the Müllerian duct epithelium (Parr and McMahon, 1998). *Wnt7a*-null males do not express *Amhr2* in the Müllerian duct mesenchyme, leading to a block in regression, resulting in the formation of a uterus (Parr and McMahon, 1998). Thus, WNT signaling lies upstream of *Amhr2* transcription in the GRN (Moses and Behringer, 2019). In addition, beta-catenin is required in the ductal mesenchyme for Müllerian duct regression during male differentiation (Kobayashi et al., 2011). These observations suggest that there may be cis elements related to WNT signaling that are required for *Amhr2* transcription.

*Amhr2* is also expressed in Sertoli, granulosa cells and smooth muscle of the uterine myometrium (Arango et al., 2008). *Amhr2* expression in these cell types have been shown to be regulated by multiple factors. A binding site for NR5A1 (also known as SF-1) located within ∼300 bp upstream of the transcription start site of the human *AMHR2* gene was shown to be required for reporter expression in the NT2/D1 teratocarcinoma cell line (De Santa Barbara et al., 1998). Knockdown of *Dmrt1* in male chick embryos caused a down-regulation of *Amhr2* (Cutting et al., 2014). BMP4 and BMP15 were shown to enhance *AMHR2* transcript levels in human and sheep ovarian granulosa cells *in vitro* (Pierre et al., 2016). This response to BMP signaling was mediated by a ∼2.2 kb region 5′ of the *AMHR2* promoter (Pierre et al., 2016). A long non-coding (lnc) RNA, *lnc-Amhr2* has been identified that enhanced *Amhr2* promoter activity in a mouse ovarian granulosa cell line, OV3121 (Kimura et al., 2017). The factors that regulate *Amhr2* transcription in Müllerian duct mesenchyme cells remain to be discovered.

### Localization of a Müllerian duct mesenchyme regulatory region for *Osx* that is responsive to AMH signaling

The BAC used to generate the *Osx-Cre* line spans 204 kb of chromosome 15, from the 3′ end of *Znf40* to the 5′ end of *Sp1*. Using the *Osx-Cre* BAC transgenic mouse line, we discovered male-specific Cre reporter gene expression in the Müllerian duct mesenchyme. Although the endogenous *Osx* gene is expressed throughout the entire male Müllerian duct mesenchyme (Mullen et al., 2018), we found that *Osx-Cre* directed reporter gene expression predominantly in the anterior Müllerian duct. It is possible that there are separate cis elements that direct anterior and posterior expression. However, these findings may be unique to this one transgenic mouse line.

The *Osx-Cherry* construct encompasses 39 kb within the 204 kb *Osx-Cre* BAC region, spanning *Osx* and intergenic sequences on both the 5′ and 3’ ends of the gene. Using the *Osx-Cherry* transgenic mouse line, we found male-specific Cherry fluorescence along the entire length of the Müllerian ducts, indicating that the 39 kb region contains a Müllerian duct mesenchyme-specific regulatory sequences. AMH signaling is necessary and sufficient for *Osx* expression in the Müllerian duct mesenchyme (Mullen et al., 2018). We found that ectopic AMH provided by an *Mt1-AMH* transgene could induce *Osx-Cherry* expression in the Müllerian duct mesenchyme of female embryos. This suggests that the 39 kb *Osx* region contains sequences that respond to AMH signaling. The sequences that direct Müllerian duct mesenchyme-specific transcription and those that mediate AMH signaling may be the same but await further study.

### Two distinct Müllerian duct mesenchyme regulatory regions

In the mouse genome, *Osx* and *Amhr2* are linked, residing on chromosome 15 within 100 kb of each other. There is one gene, namely *Sp1,* located between *Osx* and *Amhr2*. *Sp1* encodes a transcription factor that is expressed in most tissues (O’connor et al., 2016). Interestingly, the 39 kb *Osx-Cherry* region (NCBI37/mm9: Chr 15, 102,182,823–102,220,102) does not overlap with the *Amhr2* BAC clone (NCBI37/mm9: Chr 15, 102,401,136–102,548,427). Therefore, the Müllerian duct mesenchyme regulatory region we identified for *Osx-Cherry* is distinct from the one identified within the *Amhr2* BAC. The identification of two distinct Müllerian duct mesenchyme regulatory regions, one for *Amhr2* and one for *Osx*, seems reasonable because *Amhr2* is expressed in the mesenchyme of both male and female Müllerian ducts that is dependent on epithelial-derived WNT7A, whereas *Osx* expression is restricted to male Müllerian ducts that depends on *Amhr2* and AMH secreted by the fetal testes (Baarends et al., 1994; Parr and McMahon, 1998; Arango et al., 2008; Mullen et al., 2018). Thus, *Amhr2* is upstream of *Osx* in the GRN. Analysis of the chromatin landscape of E14.5 male and female Müllerian duct mesenchyme could point to Müllerian duct-specific regulatory elements. However, these types of studies have not been reported perhaps because there are very few cells per embryo and single cell approaches still require cell numbers greater than practically available. The identification of genomic regions that contain regulatory sequences for Müllerian duct mesenchyme expression contributes to the definition of this GRN of sexual development (Moses and Behringer, 2019).

## Data Availability

The original contributions presented in the study are included in the article/Supplementary Material, further inquiries can be directed to the corresponding authors.
