# Supplementary material for: A transgenic bacterial artificial chromosome approach to identify regulatory regions that direct Amhr2 and Osterix expression in Müllerian duct mesenchyme
Source: Front Cell Dev Biol. 2022 Oct 12;10:1006087. doi: 10.3389/fcell.2022.1006087 (PMC9597298; doi:10.3389/fcell.2022.1006087)
Supplement: Supplementary file 1 [file Image1.PDF]

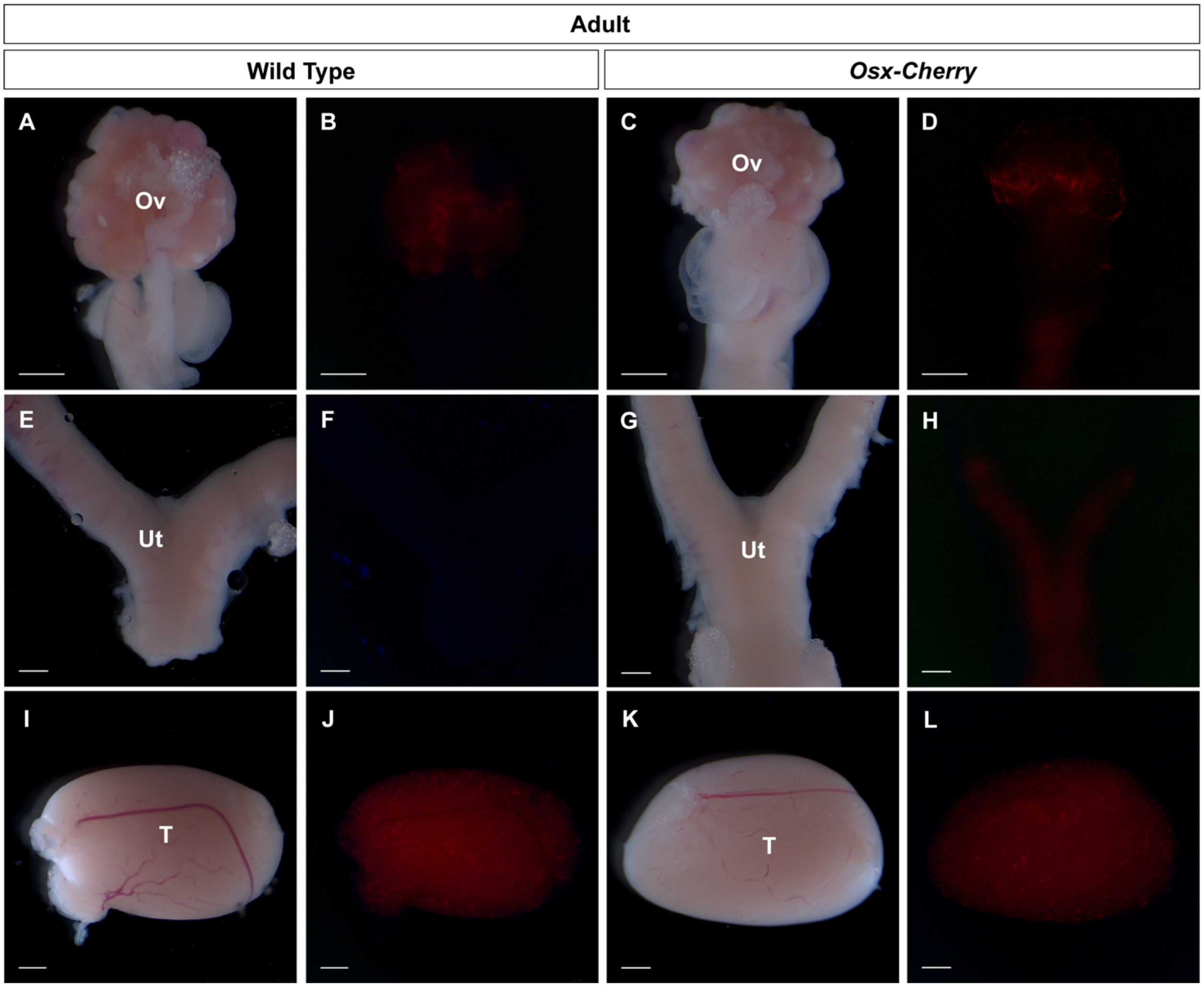

Supplemental Fig. 1. ***Osx-Cherry*** **expression in adult organs.** (**A-D**) Whole mount images of adult ovaries, (**E-H**) adult uterus, and (**I-L**), adult testis. (**A, C, E, G, I, K**) Brightfield and (**B, D, F, J, L**) fluorescent images. (**A, B, E, F, I, J**) Wild type, (**C, D, G, H, K, L**) *Osx-Cherry*. Scale bars = 1000 um. N=3 for each sex and genotype.
